# The complete chloroplast genome sequence of *Rosa laevigata* (Rosaceae)

**DOI:** 10.1080/23802359.2019.1674200

**Published:** 2019-10-15

**Authors:** Cheng Zhang, Xianhua Xiong, Xinfen Gao

**Affiliations:** a Chengdu Institute of Biology, Chinese Academy of Sciences, Chengdu, Sichuan, China;; b University of Chinese Academy of Sciences, Beijing, China;; c Key Laboratory of Bio-Resources and Eco-Environment of Ministry of Education, College of Life Sciences, Sichuan University, Chengdu, Sichuan, China

**Keywords:** *Rosa laevigata*, chloroplast genome, phylogenetic analyses

## Abstract

The complete chloroplast genome of *Rosa laevigata* has been characterized by reference-based assembly using Illumina paired-end data. The complete cp genome is 156,342 bp in length, containing a large single-copy region of 85,459 bp, a small single-copy region of 18,785 bp, which are separated by a pair of inverted repeat regions of 26,049 bp, a total of 140 genes were predicted from the cp genome, including 87 protein-coding genes, 39 tRNA genes, and 8 rRNA genes . Most genes occur as a single copy, while 19 gene species are duplicated. Phylogenetic analysis reveals that *R. laevigata* and *Rosa roxburghii* f. *normalis* are more closely related to each other than either is to *Rosa banksiae* var. *normalis*.

*Rosa laevigata* Michx. is the only species in *Rosa* sect. *Laevigatae*, distributed in East and South China, at altitudes of 200–1600 m. With large white fragrant flowers and glabrous leathery leaves, *R. laevigata* might make a good ornamental. Besides, its root bark contains tannin used for tanning; its fruit contains sugar used for fermenting wine; its roots, leaves, and fruit are all used medicinally (Gu 2003). In this study, we first reported the complete chloroplast (cp) genome of *R. laevigata*.

A wild individual of *R. laevigata* was sampled from Hangzhou City, Zhejiang Province, East China. The voucher specimen was deposited in the Herbarium of Chengdu Institute of Biology (CDBI: LLS0004-1). The total genomic DNA was extracted from leaves dried by silica gel using a modified CTAB method (Doyle and Doyle 1987) and sequenced based on the Illumina pair-end technology. The filtered reads were assembled using the programme NOVOPlasty (Dierckxsens et al. 2017) with complete cp genome of *Rosa ordoata* var. *gigantea* as the reference (GenBank accession No. KF753637). The assembled cp genome was annotated using Plann v.1.1 (Huang and Cronk 2015), and the annotation result was modified using Geneious v.10.2.3 (Kearse et al. 2012). The sequences were aligned using the MAFFT (Katoh and Standley 2013) using the published plastid genomes (excluding rose hybrids and cultivated species). Poorly aligned regions were trimmed using Gblocks v.0.91b (Castresana 2000) with the option ‘−t = c’ (the type of sequence was set to codons). The maximum-likelihood (ML) tree was constructed using RAxML v.8.2.11 (Stamatakis 2006) with 1000 bootstrap replicates to examine the phylogenetic position of *R. laevigata* in *Rosa* genus.

The complete cp genome of *R. laevigata* (Genbank accession No. MN372205) was a circular molecular genome with a size of 156,342 bp in length, which presented a typical quadripartite structure containing two inverted repeat (IR) regions of 26,059 bp each, separated by the large single-copy (LSC) region of 85,459 bp, and small single-copy (SSC) region of 18,785 bp. The overall GC content was about 37.3%. The cp genome consists of 140 genes including 87 protein-coding genes, 39 tRNA genes, and 8 rRNA genes (4 rRNA species). Most genes occur as a single copy, while 19 gene species are duplicated. Our phylogenetic result (Figure 1) shows that *R. laevigata* and *Rosa roxburghii* f. *normalis* are more closely related to each other than either is to *Rosa banksiae* var. *normalis*. The clade of the three rose species has short internal branch length with lower support values, which may be caused by short-term radiation evolution and they form a sister group to the clade composed of *Rosa* sect. *Chinensis* and *Rosa* sect. *Synstylae*.

**Figure 1.**
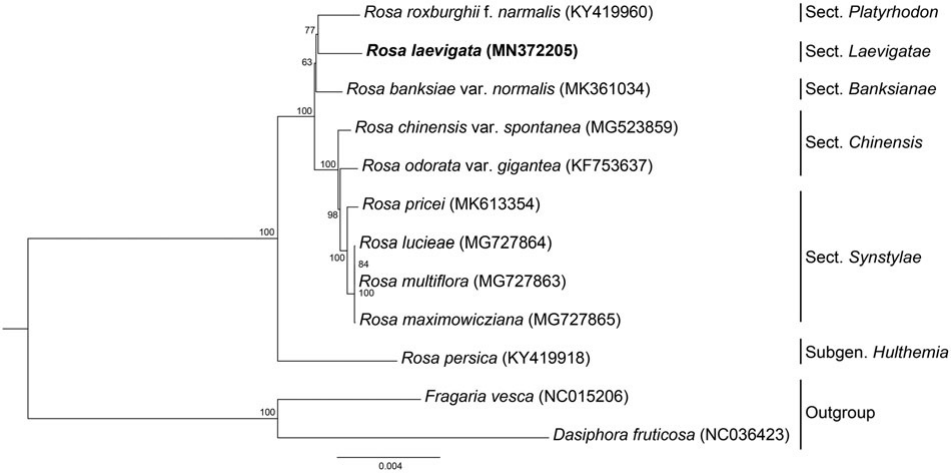
Phylogeny of chloroplast (cp) genome dataset, maximum-likelihood bootstrap support values are shown along the branches.
